# Developing a scoring tool to estimate the risk of deterioration for normotensive patients with acute pulmonary embolism on admission

**DOI:** 10.1186/s12931-020-01602-x

**Published:** 2021-01-06

**Authors:** Yizhuo Gao, Chao Ji, Hongyu Zhao, Jun Han, Haitao Shen, Dong Jia

**Affiliations:** 1 Department of Pulmonary and Critical Care Medicine, Shengjing Hospital of China Medical University, No. 36, Sanhao Street, Shenyang, China; 2 Department of Clinical Epidemiology, Shengjing Hospital of China Medical University, No. 36, Sanhao Street, Shenyang, China; 3 Department of Emergency Medicine, Shengjing Hospital of China Medical University, No. 36, Sanhao Street, Shenyang, China

**Keywords:** Pulmonary embolism, Computerized tomography, Nomogram, Right ventricle

## Abstract

**Background:**

It is important to identify deterioration in normotensive patients with acute pulmonary embolism (PE). This study aimed to develop a tool for predicting deterioration among normotensive patients with acute PE on admission.

**Methods:**

Clinical, laboratory, and computed tomography parameters were retrospectively collected for normotensive patients with acute PE who were treated at a Chinese center from January 2011 to May 2020 on admission into the hospital. The endpoint of the deterioration was any adverse outcome within 30 days. Eligible patients were randomized 2:1 to derivation and validation cohorts, and a nomogram was developed and validated by the aforementioned cohorts, respectively. The areas under the curves (AUCs) with 95% confidence intervals (CIs) were calculated. A risk-scoring tool for predicting deterioration was applied as a web-based calculator.

**Results:**

The 845 eligible patients (420 men, 425 women) had an average age of 60.05 ± 15.43 years. Adverse outcomes were identified for 81 patients (9.6%). The nomogram for adverse outcomes included heart rate, systolic pressure, N-terminal-pro brain natriuretic peptide, and ventricle/atrial diameter ratios at 4-chamber view, which provided AUC values of 0.925 in the derivation cohort (95% CI 0.900–0.946, *p* < 0.001) and 0.900 in the validation cohort (95% CI 0.883–0.948, *p* < 0.001). A risk-scoring tool was published as a web-based calculator (https://gaoyzcmu.shinyapps.io/APE9AD/).

**Conclusions:**

We developed a web-based scoring tool that may help predict deterioration in normotensive patients with acute PE.

## Background

In 2019, the European Society of Cardiology (ESC) revised the risk stratification system for patients with acute pulmonary embolism (PE) based on the 2014 ESC guideline [1, 2]. However, 2019 ESC guidelines still emphasized the critical role of identifying patients with poor prognosis from normotensive patients by the Bova and Fast scores [1]. Furthermore, there are patients with poor prognosis who have shown a false sense of security with normotension at their admission, which might mask the risk of rapid deterioration and death [3, 4]. Unfortunately, there is no universally recognized tool for distinguishing these patients and guiding clinical decision-making to define the appropriate treatment strategy [1].

Computed tomography (CT) pulmonary angiography can be used to diagnose PE and also can be used for identifying patients who have poor prognosis [5–7]. However, most models for predicting a poor short-term prognosis among normotensive patients with acute PE have not incorporated CT parameters or only used a CT parameter for identifying right ventricle (RV) dysfunction [3, 8–10]. Different measurement methods and different thresholds in the evaluation of right-to-left heart size by CT caused this discrepancy [11, 12]. Therefore, redefining the ratio of right-to-left heart size parameters from CT and then combining clinical and laboratory parameters might help promote predictive ability [1].

Although CT parameters regarding cardiac size can reflect an increased impedance in the pulmonary circulation [13]. Individual responses to the right ventricular afterload increase varies broadly depending on comorbidities, associated vasoconstriction, degree of proximal obstruction, and RV straining. These broad variations can be manifested in clinical and laboratory parameters, such as hypotension, tachycardia, myocardial markers and ultrasound RV dysfunction [1]. Based on the 2019 ESC guideline, the present study aimed to develop a semi-quantitative tool that combined clinical, laboratory, and simple CT parameters to promote predictive ability in normotensive patients with acute PE poor prognosis.

## Methods

### Study design

This retrospective study evaluated clinical, laboratory, and simple CT parameters of normotensive patients with acute PE from admission. The Bova score and 2019 ESC algorithm were used for risk stratification. The outcomes of interest were defined as the occurrence of adverse outcomes within 30 days after admission into hospital. Eligible patients were randomized 2:1 into derivation and validation cohorts. The derivation cohort was used to develop and evaluate a multivariable logistic regression model for predicting the outcomes of interest. The discriminatory power was evaluated by comparing the nomogram to the established risk stratification systems. The consistency of the nomogram was evaluated using the validation cohort. The investigators independently collected the data regarding clinical, laboratory, and CT parameters as well as data regarding the risk stratification scores and outcomes of interest. This research was approved by the Institutional Review Board of the Shengjing Hospital of China Medical University (No. 2020PS522K), and informed consent was exempted due to the absence of treatment intervention in patients.

### Patient selection

Normotensive patients with acute PE were evaluated if they were treated at the Shengjing Hospital of China Medical University between January 2011 and May 2020. The diagnosis and management of acute PE was based on the 2019 ESC guidelines [1]. The inclusion criteria were an age of ≥ 18 years and a PE diagnosis based on CT pulmonary angiography. The exclusion criteria were pregnancy, reception of reperfusion treatment before admission, and missing data regarding CT parameters, echocardiography, cardiac troponin I (c-Tn I), and N-terminal-pro brain natriuretic peptide (NT-pro BNP) levels.

### Clinical data

The patients’ medical records were reviewed to collect their demographic characteristics and baseline data from their admission regarding heart rate, systolic pressure, history of disease, arterial oxyhemoglobin saturation, c-Tn I concentration (μg/L), and NT-pro BNP concentration (pg/mL).

### Assessing RV dysfunction

Within 24 h after admission, RV dysfunction determined a transthoracic echocardiography using an IE Elite ultrasound machine (Philips) equipped with an S 5–1 transducer (frequency conversion 1–5 MHz) by ultrasound specialist as following criteria: RV dilation (end-diastolic diameter > 30 mm, evaluated at 4-chamber view or parasternal view), an increased RV/left ventricle(LV) end-diastolic diameter ratio > 0.9 at 4-chamber view, hypokinesia of the free RV wall, increased velocity of the jet of tricuspid regurgitation at apical 4-chamber view, decreased tricuspid annulus plane systolic, anyone or combinations of the condition above [2, 14].

### Risk stratification

Risk stratification was based on the 2019 ESC algorithm [1] and Bova score [10], with classifications as “low risk,” “intermediate-low risk,” and “intermediate-high risk” (Additional file 1: Table S1 and Additional file 2: Table S2). The 2019 ESC algorithm evaluated c-Tn I (cutoff: 0.04 μg/L), NT-pro BNP (cutoff: 600 pg/mL) levels, RV dysfunction, and the simple PE severity index [1]. The Bova score was calculated based on c-Tn I (cutoff: 0.05 µg/L), RV dysfunction, heart rate (cutoff: 110 beats/min), and systolic pressure (cutoff: 90–100 mmHg).

### Outcomes of interest

The outcomes of interest were defined as the occurrence of adverse outcomes within 30 days after admission. Adverse outcomes were defined as PE-related deaths, the need for mechanical ventilation, the need for cardiopulmonary resuscitation, and the need for life-saving vasopressor and reperfusion treatment [9, 15].

### Measurement of CT parameters

Three simple CT parameters were selected for the analysis. The first factor was thrombus location, which was categorized as within the central pulmonary artery (CPA embolism) [15, 16], spanning both sides of the bifurcation (saddle-CPA embolism) [17], and outside the CPA (non-CPA embolism) (Additional file 3: Figure S1 a–c). The second factor was the RV and LV diameters in the short-axis plane, which were measured as the maximal diameter from the cardiac intima to the interventricular septum [18], as well as the relative ratio of the RV/LV short-axis diameters (Additional file 4: Figure S2). The third factor was the maximum chamber diameters, which were measured using a 4-chamber view perpendicular to the atrial and interventricular septum (Additional file 4: Figure S2)[6], as well as the relative ratios of the RV/LV and right atrium (RA)/left atrium (LA) 4-chamber diameters. All CT parameters were measured using the Mimics Medical software (version 19.0, Mimics Medical software, Leuven, Belgium).

### Development of the model and risk-scoring tool

The model was developed based on three steps: (a) identifying relevant prognostic factors; (b) developing and validating the model; (c) evaluating the model’s discriminatory power relative to the 2019 ESC algorithm and Bova score.

In the first step, eligible patients were randomized 2:1 into derivation and validation cohorts based on the TRIPOD standard [19]. All clinical, laboratory, and CT parameters were included into a classification and regression tree (CART) to identify relevant prognostic factors with importance [20]. All potential decisional factors for adverse outcomes were evaluated and chosen into splits providing the optimal separations by binomial data until the splits reached a minimum size or no improvement could be made [21]. All the chosen binomial parameters from CART were used to develop the model.

In the second step, univariate and multivariate logistic regression analyses were used to investigate binomial prognostic factors using the derivation cohort, and a nomogram was created by converting each regression coefficient from the multivariate logistic regression onto a scale of 0 points (low) to 100 points (high). The total scores for all variables were summed [22], and the different risk groups were separated based on their total nomogram scores via another CART analysis. A validation cohort was used to evaluate the model’s consistency relative to the observed outcomes [21]. A calibration curve was used to assess the consistency between actual incidence and predicted incidence of the nomogram in the derivation and validation cohorts.

In the third step, the models’ abilities to predict adverse outcomes were compared to the 2019 ESC algorithm [1] and the Bova score [10] based on the receiver-operating characteristic curve (ROC) and decision curve analysis (DCA). The final risk-scoring tool was published as a free web-based calculator.

### Statistical analysis

Continuous variables were expressed as mean ± standard deviation and compared using the Student’s t test. Categorical variables were presented as numbers (%) and compared using the χ^2^ test. By a recursive partitioning analysis, CART was used to dichotomize each variable while controlling for confounders and divide the derivation cohort into different risk groups according to the total nomogram score [23]. Univariate and multivariate logistic regression analyses were used to evaluate the different factors, and the results were expressed as odds ratios (ORs) with corresponding 95% confidence intervals (CIs). The nomograms’ predictive performances were evaluated based on the concordance index (C-index) and calibration with 1000 bootstrap resampling [22]. The ROC curves were used to evaluate sensitivity, specificity, positive predictive value (PPV), negative predictive value (NPV), and the area under the curve (AUC). A calibration curve was used to assess the consistency between the actual incidence and predicted incidence of the nomogram [24]. Clinical utility was evaluated based on net benefit from the DCA. DeLong’s test was used to compare AUC values [25]. Differences were considered significant at *p*-values of < 0.05, and all analyses were performed using R software (version 4.0.1; R Foundation, https://www.r-project.org).

## Results

### Demographics and baseline characteristics

**Fig. 1:**
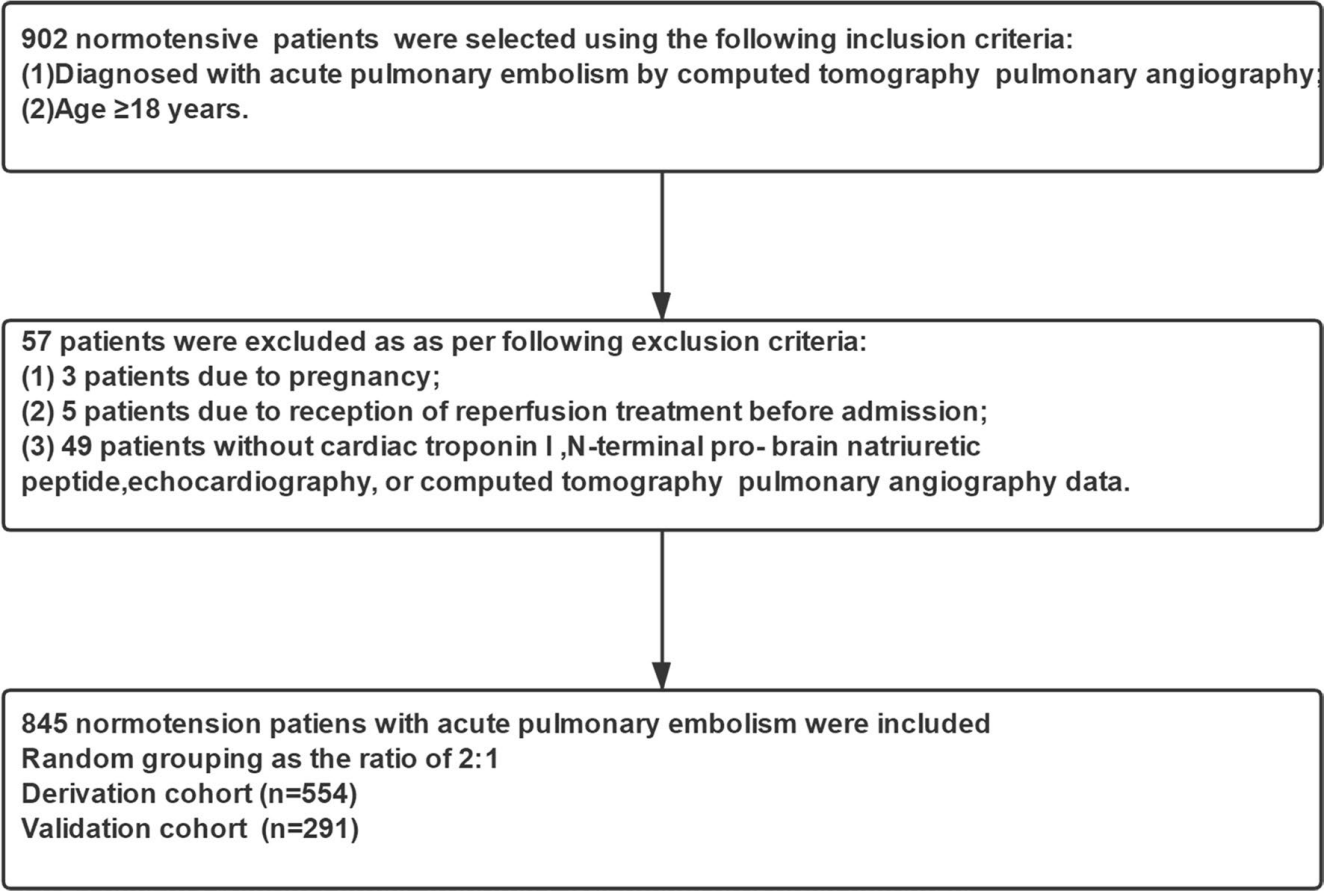
Flowing chart of inclusion and exclusion criteria

We evaluated 902 normotensive patients with acute PE, although 57 patients were excluded; a total of 3 patients were excluded due to pregnancy, 5 patients were excluded due to reception of reperfusion treatment, and 49 patients were excluded due to the absence of data on CT parameters, echocardiography, c-Tn I, or NT-pro BNP. Finally, a total of 845 eligible patients were included (Fig. 1), including 420 male patients and 425 female patients with an average age of 60.05 ± 15.43 years. Adverse outcomes were identified for 81 patients (42 male and 39 female) who had an average age of 59.36 ± 15.74 years (Table 1). No adverse outcomes were identified for 764 patients (378 male and 386 female) who had an average age of 60.12 ± 15.40 years. Patients with adverse outcomes had significantly higher values for heart rate, c-Tn I, and NT-pro BNP (all *p* < 0.001) and had significantly lower value of systolic pressure (*p* < 0.001). Patients with adverse outcomes were more likely to have RV dysfunction (*p* < 0.001). Among CT parameters, patients with adverse outcomes were more likely to have CPA saddle-CPA embolisms (both *p* < 0.001). Furthermore, patients with adverse outcomes had high values of the RV short-axis diameter, RV 4-chamber diameter, RA 4-chamber diameter, RV/LV short-axis diameter ratio, RV/LV 4-chamber diameter ratio, and RA/LA 4-chamber diameter ratio (all *p* < 0.001). However, patients with adverse outcomes also had lower values for the LV short-axis diameter, LV 4-chamber diameter, and LA 4-chamber diameter (all *p* < 0.001).

**Table 1:** Baseline characteristics among patients with and without adverse outcomes

	All patients	Adverse outcomes		*p-*value
	(n = 845)	Yes (n = 81)	No (n = 764)	
Sex (male)	420 (49.7%)	42(51.9%)	378 (49.5%)	0.68
Age (years)	60.05 ± 15.43	59.36 ± 15.74	60.12 ± 15.40	0.93
Heart rate (beats/min)	86.57 ± 17.81	108.64 ± 22.84	84.23 ± 15.45	< 0.001
Systolic pressure (mmHg)	124.33 ± 18.26	115.22 ± 19.64	125.30 ± 17.85	< 0.001
RV dysfunction	240(28.4%)	59(72.8%)	181(23.7%)	< 0.001
c-Tn I (*μ*g/L)	0.11 ± 0.47	0.25 ± 0.49	0.091 ± 0.46	< 0.001
NT-pro BNP (*p*g/mL)	1,547.12 ± 3,652.06	131,039 ± 3,255.93	3,779.92 ± 5,832.71	< 0.001
CPA embolism	209 (24.7%)	45 (55.6%)	164 (22.0%)	< 0.001
Saddle-CPA embolism	62 (7.3%)	17 (21.0%)	45 (5.9%)	< 0.001
RV short-axis diameter (mm)	39.21 ± 7.42	44.70 ± 8.53	38.62 ± 7.05	< 0.001
LV short-axis diameter (mm)	40.76 ± 7.30	33.68 ± 6.91	41.51 ± 6.94	< 0.001
RV4-chamber diameter (mm)	36.38 ± 7.37	42.64 ± 10.28	35.72 ± 6.67	< 0.001
LV 4-chamber diameter (mm)	39.47 ± 7.36	31.63 ± 7.09	40.30 ± 6.88	< 0.001
RA 4-chamber diameter (mm)	45.44 ± 9.34	52.38 ± 11.72	44.70 ± 8.74	< 0.001
LA 4-chamber diameter (mm)	34.83 ± 8.52	29.18 ± 8.00	35.43 ± 8.35	< 0.001
RV/LV short-axis diameter ratio	1.00 ± 0.30	1.38 ± 0.40	0.96 ± 0.25	< 0.001
RV/LV 4-chamber diameter ratio	0.96 ± 0.31	1.44 ± 0.59	0.90 ± 0.20	< 0.001
RA/LA 4-chamber diameter ratio	1.39 ± 0.52	1.97 ± 0.87	1.33 ± 0.42	< 0.001
Bova score				
Low risk	681 (75.9%)	33 (40.7%)	648 (84.4%)	< 0.001
Intermediate-low risk	102 (12.1%)	17 (20.7%)	85 (11.1%)	0.009
Intermediate-high risk	62 (7.3%)	31 (38.3%)	31 (4.1%)	< 0.001
2019 ESC algorithm				
Low risk	503 (59.5%)	17 (21.0%)	486 (63.6%)	< 0.001
Intermediate-low risk	172 (20.4%)	14 (17.3%)	158 (20.7%)	0.47
Intermediate-high risk	170 (20.1%)	50 (61.7%)	120 (15.7%)	< 0.001

*c Tn-I* cardiac troponin I, *NT-pro BNP* N-terminal pro-brain natriuretic peptide, *CPA* central pulmonary artery, *RV* right ventricle, *LV* left ventricle, *RA* right atrium, *LA* left atrium, *ESC* European Society of Cardiology

The Bova score for patients with adverse outcomes revealed low risk (33 patients), intermediate-low risk (17 patients), and intermediate-high risk (31 patients). The Bova score for patients without adverse outcomes revealed low risk (648 patients), intermediate-low risk (85 patients), and intermediate-high risk (31 patients). The 2019 ESC algorithm for patients with adverse outcomes revealed low risk (17 patients), intermediate-low risk (14 patients), and intermediate-high risk (50 patients). The 2019 ESC algorithm for patients without adverse outcomes revealed low risk (486 patients), intermediate-low risk (158 patients), and intermediate-high risk (120 patients) (Table 1).

### Comparison between derivation and validation cohorts

After a random grouping with a ratio of 2:1, a total of 554 and 291 patients were divided into derivation and validation cohorts, respectively. In the derivation cohort, the average age was 60.43 ± 14.95 years including 267 men and 287 women. In the validation cohort, the average age was 59.32 ± 16.30 years including 153 men and 138 women. There was no statistical difference between derivation and validation cohorts (all *p* > 0.05) (Table 2).

**Table 2:** Comparation between derivation and validation cohorts

	Derivation cohort (n = 554)	Validation cohort (n = 291)	*p*-value
Sex (male)	267 (48.2%)	153 (52.6%)	0.23
Age (years)	60.43 ± 14.95	59.32 ± 16.30	0.33
Heart rate (beats/min)	86.11 ± 17.76	87.46 ± 17.89	0.30
Systolic pressure (mmHg)	126.92 ± 18.27	125.30 ± 18.09	0.22
RV dysfunction	161 (29.0%)	79 (27.1%)	0.56
c-Tn I (*μ*g/L)	0.11 ± 0.54	0.10 ± 0.29	0.79
NT-pro BNP (*p*g/mL)	1638.25 ± 3958.77	1373.61 ± 2980.96	0.28
CPA embolism	141 (25.5%)	68 (23.4%)	0.50
Saddle-CPA embolism	38 (6.9%)	24 (8.2%)	0.46
RV short-axis diameter (mm)	39.26 ± 7.605	39.11 ± 6.97	0.77
LV short-axis diameter (mm)	40.58 ± 7.65	41.08 ± 7.52	0.35
RV4-chamber diameter (mm)	36.22 ± 7.21	36.68 ± 7.68	0.41
LV 4-chamber diameter (mm)	39.50 ± 7.26	39.41 ± 7.55	0.86
RA 4-chamber diameter (mm)	45.50 ± 9.38	45.32 ± 9.27	0.79
LA 4-chamber diameter (mm)	34.95 ± 8.38	34.61 ± 8.77	0.58
RV/LV short-axis diameter ratio	1.00 ± 0.31	0.98 ± 0.27	0.34
RV/LV 4-chamber diameter ratio	0.95 ± 0.28	0.97 ± 0.35	0.37
RA/LA 4-chamber diameter ratio	1.39 ± 0.52	1.40 ± 0.51	0.76
Bova score			
Low risk	445 (80.3%)	236 (81.1%)	0.79
Intermediate-low risk	66 (11.9%)	36 (12.3%)	0.85
Intermediate-high risk	43 (7.8%)	19 (6.5%)	0.51
2019 ESC algorithm			
Low risk	330 (59.6%)	173 (59.5%)	0.97
Intermediate-low risk	112(20.2%)	60 (20.6%)	0.89
Intermediate-high risk	112(20.2%)	58 (19.9%)	0.92
Adverse outcomes	54(9.7%)	27(9.3%)	0.83

*c Tn-I* cardiac troponin I, *NT-pro BNP* N-terminal pro-brain natriuretic peptide, *CPA* central pulmonary artery, *RV* right ventricle, *LV* left ventricle, *RA* right atrium, *LA* left atrium, *ESC* European Society of Cardiology

### Variable selection

Five variables were considered significant predictors of adverse outcomes and were dichotomized: heart rate (≥ 110 beats/min vs. < 110 beats/min), systolic pressure (90–100 mmHg vs. > 100 mmHg), NT-pro BNP (≥ 800 pg/mL vs. < 800 pg/mL), RV/LV 4-chamber diameter ratio (≥ 1.25 vs. < 1.25), and RA/LA 4-chamber diameter ratio (≥ 1.30 vs. < 1.30). A multivariate logistic regression analysis using the derivation cohort revealed that adverse outcomes were independently predicted by heart rate (OR 7.07, 95% CI 2.92–17.09, *p* < 0.001), systolic pressure (OR 7.68, 95% CI 1.57–37.58, *p* < 0.001), NT-pro BNP (OR 3.35, 95% CI 1.36–9.17, *p* < 0.001), RA/LA 4-chamber diameter ratio (OR 3.53, 95% CI 1.27–2.85, *p* < 0.001), and RV/LV 4-chamber diameter ratio (OR 29.86, 95% CI 11.34–78.61, *p* < 0.001) (Table 3).

**Table 3:** Univariate and multivariate logistic regression analyses for developing the nomogram to predict adverse outcomes in the derivation cohort

	Univariate analysis		Multivariate analysis	
	OR (95% CI)	*p-*value	OR (95% CI)	*p-*value
Heart rate (≥ 110 vs. < 110 beats/min)	20.58 (10.77–39.33)	< 0.001	7.07 (2.92–17.09)	< 0.001
Systolic pressure (90–100 vs. > 100 mmHg)	26.11 (9.43–72.28)	< 0.001	7.68 (1.57–37.58)	0.012
NT-pro BNP (≥ 800 vs. < 800 pg/mL)	7.94 (4.07–15.50)	< 0.001	3.35 (1.39–8.11)	0.0073
RV/LV 4-chamber diameter ratio (≥ 1.25 vs. < 1.25)	64.66 (28.84–144.96)	< 0.001	29.86 (11.34–78.61)	< 0.001
RA/LA 4-chamber diameter ratio (≥ 1.30 vs. < 1.30)	6.63 (3.17–13.85)	< 0.001	3.53 (1.36–9.17)	0.0096

*NT-pro BNP* N-terminal pro-brain natriuretic peptide; *RV* right ventricle; *LV* left ventricle; *RA* right atrium; *LA* left atrium; *OR* odds ratio; *CI* confidence interval

### Performance of the nomograms in the derivation and validation cohorts

**Fig. 2:**
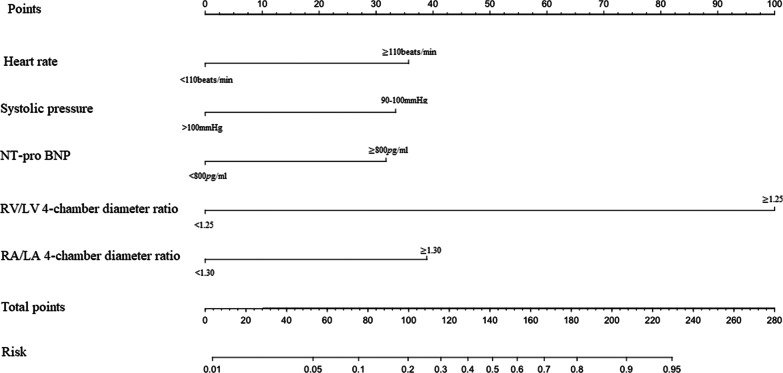
The nomogram for predicting the risk of adverse outcomes. *c Tn-I* cardiac troponin I; *NT-pro BNP* N-terminal pro-brain natriuretic peptide; *RV* right ventricle; *LV* left ventricle; *RA* right atrium; *LA* left atrium

**Fig. 3:**
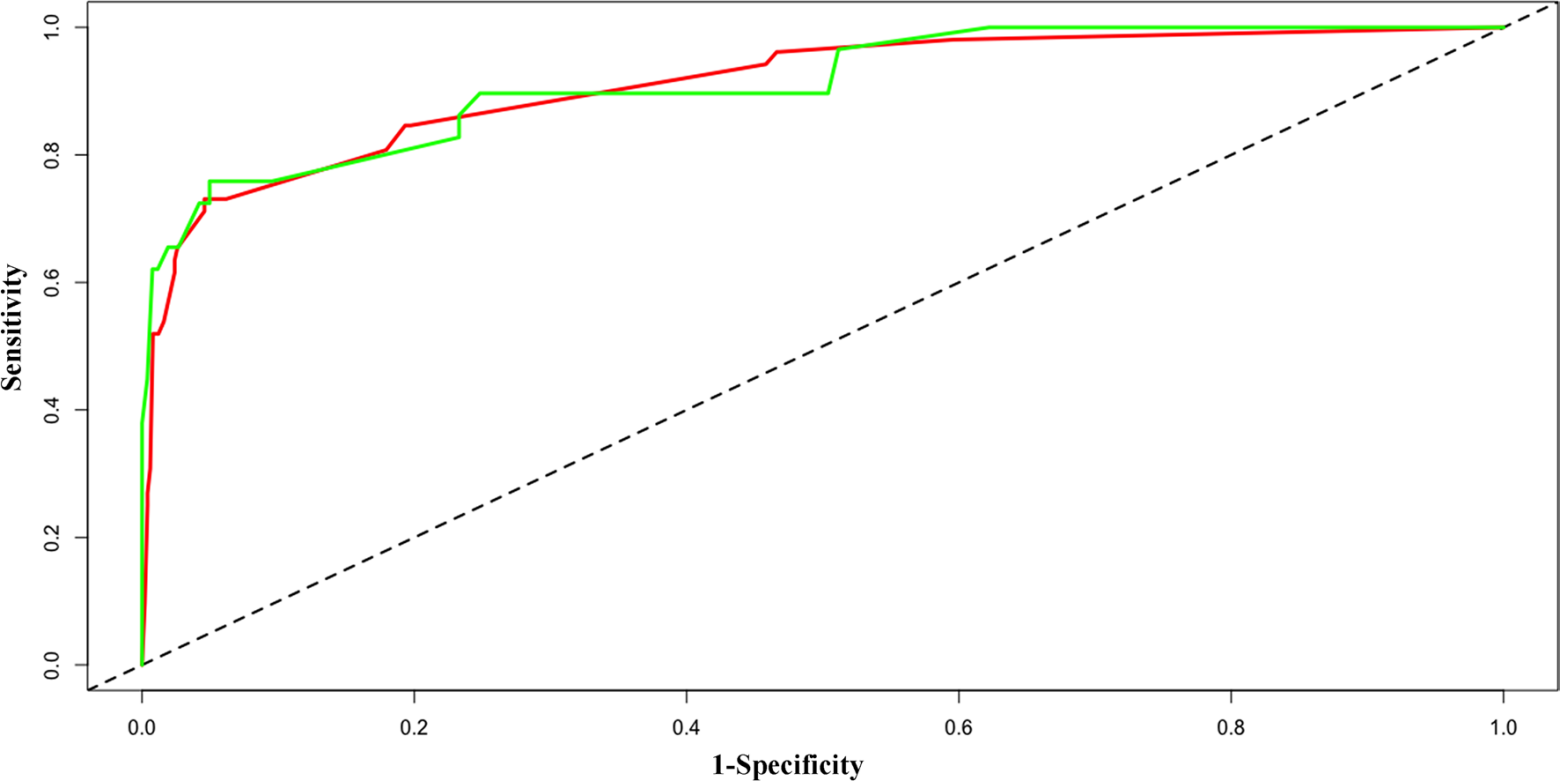
The receiver-operating characteristic curves for predicting adverse outcomes. The training dataset is shown using the red line and the validation dataset is shown using the green line. The area under the curve values for predicting adverse outcomes were 0.925 in the training dataset (95% confidence interval [CI] 0.900–0.946, *p* < 0.001) and 0.900 in the validation dataset (95% CI 0.883–0.948, *p* < 0.001)

**Fig. 4:**
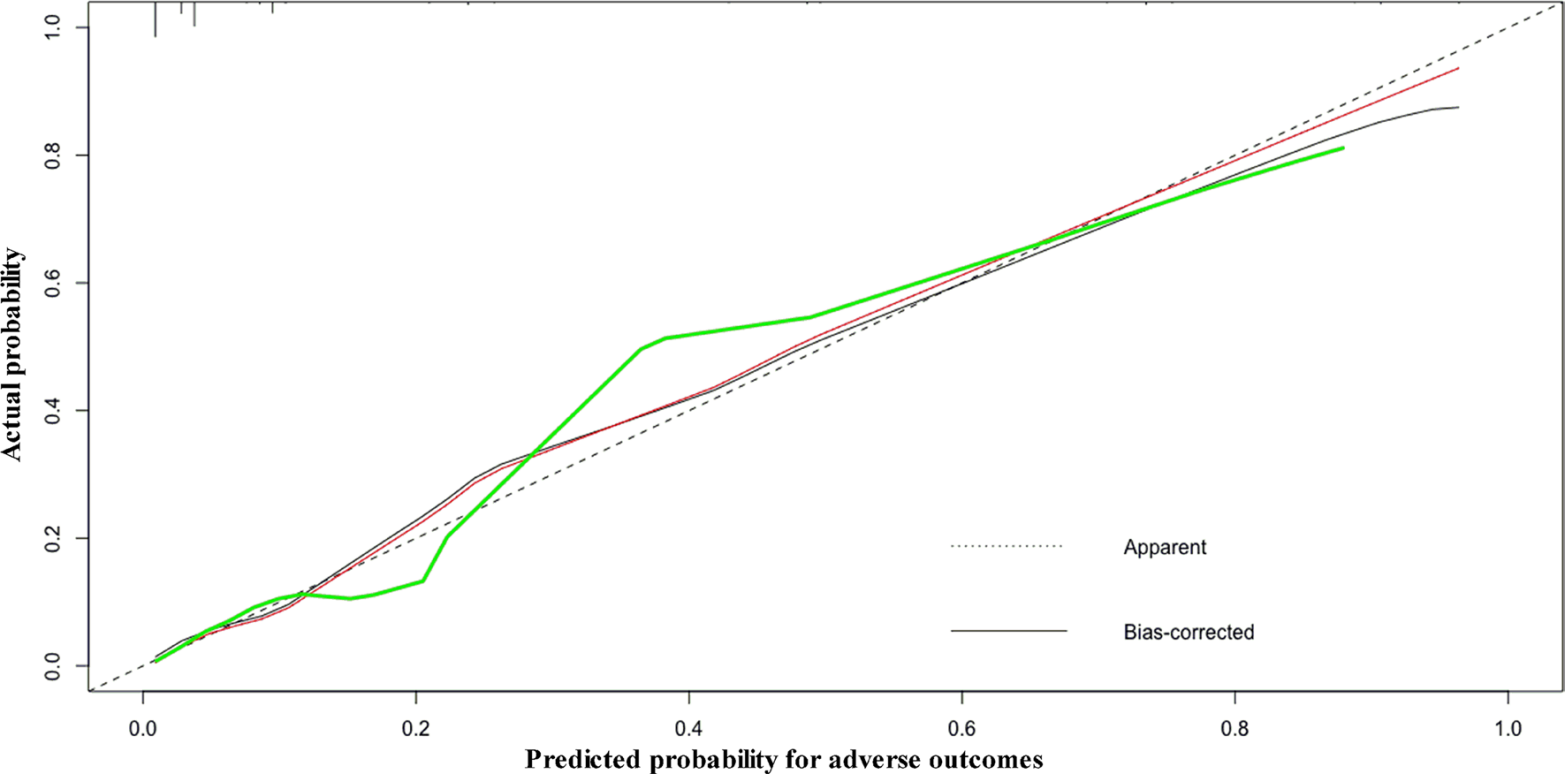
Calibration curves for the nomogram predicting adverse outcomes. The training dataset is shown using the red line, and the validation dataset is shown using the green line

Nomograms were developed using the two multivariate logistic regression models (Fig. 2). The nomogram for predicting adverse outcomes incorporated five variables and provided good C-index values in the derivation (C-index: 0.925, 95% CI 0.900–0.946) and validation cohorts (C-index: 0.900, 95% CI 0.883–0.948) (Fig. 3). The calibration curve also revealed good agreement between the nomogram’s predictions and the actual outcomes (Fig. 4).

### Predicting adverse outcomes based on the nomogram, Bova score, and 2019 ESC algorithm

The AUC values for predicting adverse outcomes were 0.925 for the nomogram (95% CI 0.900–0.946, *p* < 0.001), 0.797 for the Bova score (95% CI 0.761–0.830, *p* < 0.001), and 0.790 for the 2019 ESC algorithm (95% CI 0.753–0.823, *p* < 0.001). Comparing the nomogram and Bova score revealed a difference in AUC values of 0.128 (95% CI 0.072–0.184, *p* < 0.001). A comparison of the nomogram and 2019 ESC algorithm revealed a difference in AUC values of 0.136 (95% CI 0.075–0.196, *p* < 0.001). The nomogram had a higher PPV for predicting adverse outcomes (66.5%) than did the Bova score (34.8%) or the 2019 ESC algorithm (31.3%) (Table 4).

**Table 4:** Comparing the nomogram to risk stratification for adverse outcomes

	AUC (95% CI)	Sensitivity (%)	Specificity (%)	PPV (%)	NPV (%)
Nomogram	0.925 (0.900–0.946)	74.1	95.1	65.5	97.2
Bova score	0.797 (0.761–0.830)	70.4	85.8	34.8	96.4
2019 ESC algorithm	0.790 (0.753–0.823)	64.8	84.6	31.3	95.7

*ESC* European Society of Cardiology, *AUC* area under the curve, *PPV* positive predictive value, *NPV* negative predictive value, *CI* confidence interval

**Fig. 5:**
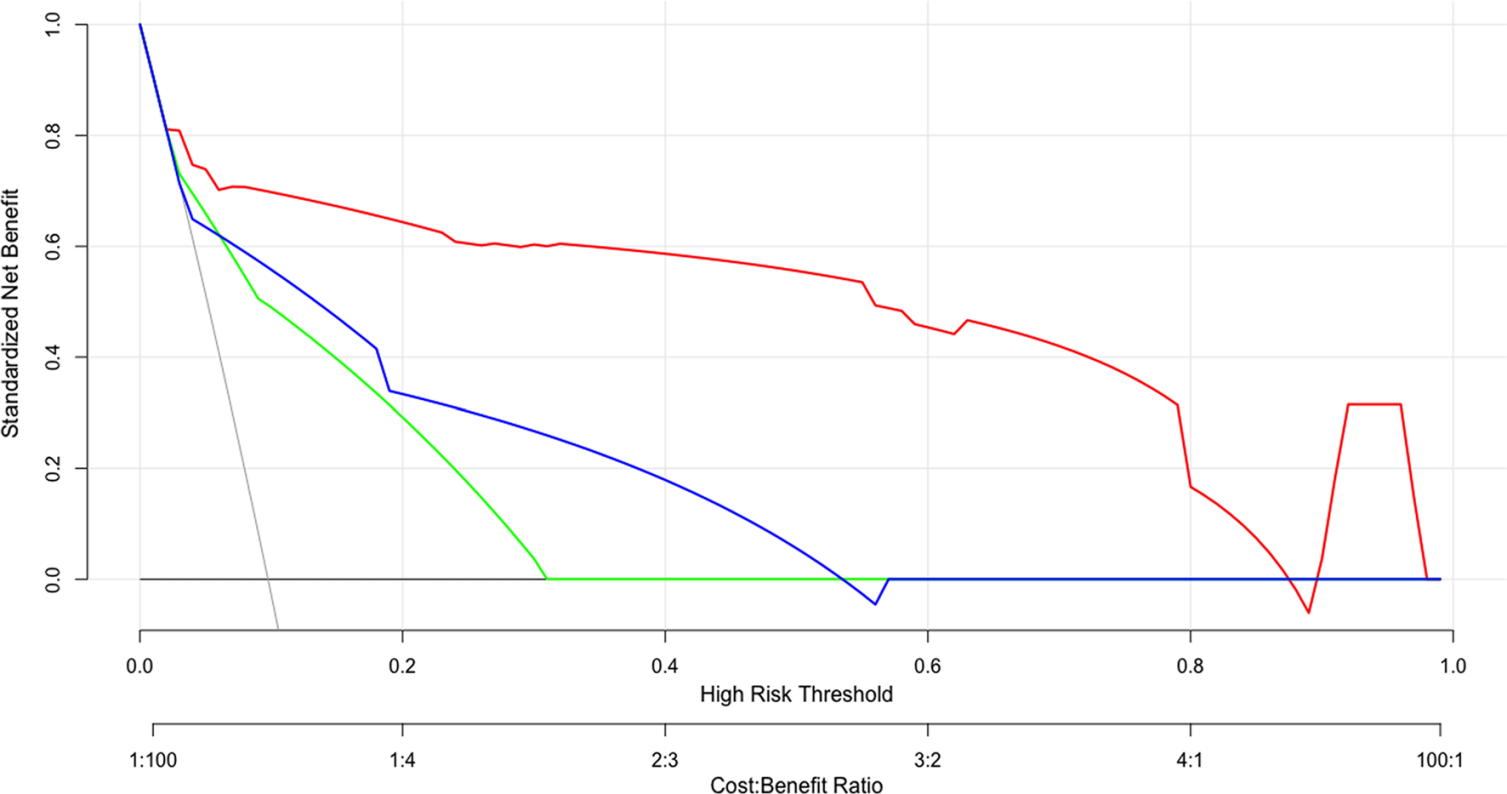
Decision curve analysis for the nomogram. A comparison of net benefit between the nomogram, the 2019 ESC algorithm, and the Bova score, which revealed that the nomogram was better in terms of net benefit (net benefit: 0.03–0.98). The nomogram is shown using the red line, Bove score is shown using the green line, and 2019 ESC algorithm is shown using green line. *ESC* European Society of Cardiology

The DCA revealed that the nomogram had greater net benefit than the 2019 ESC algorithm or the Bova score for predicting adverse outcomes. Using the nomogram for predicting adverse outcomes added a net benefit of 0.03–0.98 (Fig. 5).

### Development of the risk-scoring tool

**Fig. 6:**
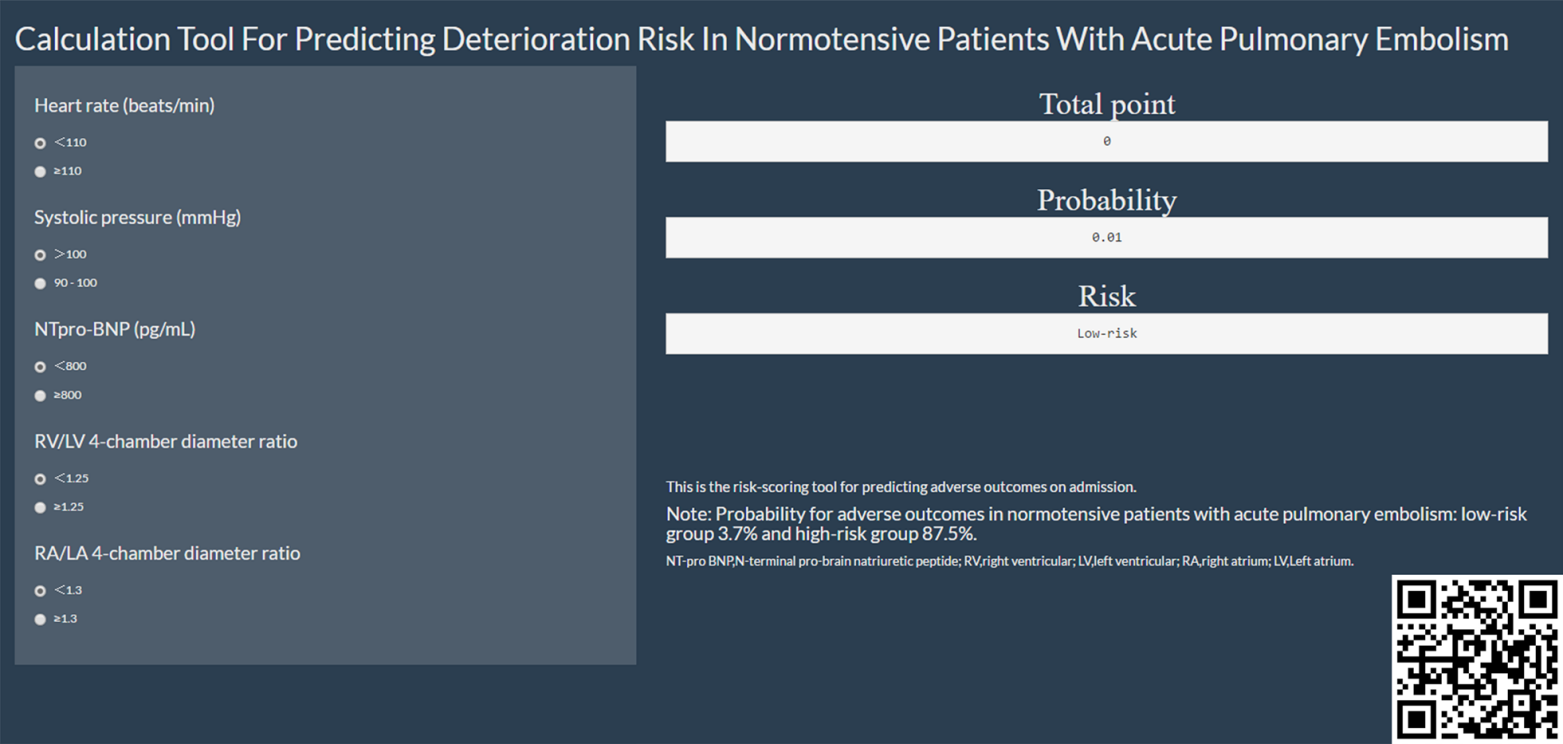
The web-based calculator for estimating the risk of adverse outcomes (https://gaoyzcmu.shinyapps.io/APE9AD/). The QR code in the lower right corner can be used to publish the result to mobile electronic equipment. *NT-pro BNP* N-terminal pro-brain natriuretic peptide; *RV* right ventricle; *LV* left ventricle; *RA* right atrium; *LA* left atrium

The nomogram for predicting adverse outcomes was used to develop a web-based calculator (https://gaoyzcmu.shinyapps.io/APE9AD/), which assigned patients to a high-risk group (≥ 145 points) or a low-risk group (< 145 points). The QR code in the lower right corner of each calculator in Fig. 6 can be used to publish the results to mobile electronic equipment.

## Discussion

This study developed a tool for estimating the risk of deterioration among normotensive patients with acute PE. Results revealed that the risk of adverse outcomes within the first 30 days after admission could be predicted using a nomogram that incorporated the RV/LV and RA/LA 4-chamber diameter ratios, NT-pro BNP concentration, systolic pressure, and heart rate. Furthermore, this risk-scoring tool had better discriminatory power and a greater net benefit than did the 2019 ESC algorithm and the Bova score. Finally, this tool was converted into convenient web-based calculators that could be used in clinical practice.

Our results are consistent with the Bova score as a decreased systolic pressure (90–100 mmHg) and an elevated heart rate (≥ 110 beats/min) were risk factors for adverse outcomes. In normotensive patients with acute PE that is deteriorating, the presentation of lower systolic pressure together with more tachycardia are warning of overt RV dysfunction [26] and are associated with the poor short-term prognosis in acute PE [1, 27]. However, we found some differences that elevated NT-pro BNP concentration was included in the final scoring tool for predicting adverse outcomes, and c-Tn I concentration was not included. c-Tn I was still one of the important predictors of adverse outcomes in patients with acute PE [1, 10]. Based on the CART analysis that selected variables that were most likely to identify adverse outcomes [28], c-Tn I might not have had enough importance compared to the other parameters in our study. In some previous studies, the elevated c-Tn I was also not included after a multifactor analysis [29, 30]. NT-pro BNP reflected RV strain, which was complementary and not mutually exclusive to CT parameters [29]. This might be the reason that the NT-pro BNP, not c-Tn I, was included into the final model in our study.

Interestingly, the increased predictive value of our risk-scoring tool (vs. the 2019 ESC algorithm and the Bova score) was mainly related to the RV/LV and RA/LA 4-chamber diameter ratios. A previous report had described the interaction between the RV and LV via the interventricular septum [31], and the RV and pulmonary circulation are characterized by low impedance and high output [11]. Furthermore, the size of the LV is larger than the size of the RV. Thus, severe PE leads to increased pressure in the RV, which compresses the LV via the interventricular septum, and clear RV-to-LV compression can be observed in cases with severe chronic pulmonary hypertension (PH) [32]. Severe PH is also the main pathophysiological mechanism underlying the occurrence of adverse outcomes in acute PE [1]. Decreased blood return to the LV also further reduces LV size, which leads to an increased right-to-left heart size ratio. The four-chamber view is defined as the plane perpendicular to the atrial and interventricular septum [6], which can be used to accurately evaluate the increased size of the right heart, the decreased size of the left heart, and the interaction between these factors. The threshold value in our study was an RV/LV 4-chamber diameter ratio of 1.25, rather than previously reported cutoffs of 0.9, 1.0, or 1.1 [33, 34], although differences in the definition of this threshold may explain the selection of different parameters between our study and previous studies. The RA/LA 4-chamber diameter ratio was another factor in our risk-scoring tool. The membranous structure [31] and greater pressure sensitivity of the atrium (vs. the ventricle) may explain the lower weighting of the RA/LA 4-chamber diameter ratio relative to the weighting of the RV/LV 4-chamber diameter ratio in our scoring tool.

## Limitations

The present study has several limitations that should be considered. First, although we evaluated data from normotensive patients with acute PE who were treated during an approximately 10-year period, the retrospective analysis is prone to bias. Second, although this scoring tool was developed using randomized derivation and validation cohorts, external validation is also required. Third, we did not have access to data regarding cardiac troponin T, and heart type fatty acid binding protein, which precluded comparisons of our tools to the fast prognostic score [9] for predicting adverse outcomes.

## Conclusion

We developed a scoring tool that was published as web-based calculators for predicting adverse outcomes among normotensive patients with acute PE. This risk-scoring tool may help improve the management of patients with acute PE by predicting deterioration.

## Supplementary information

**Additional file 1: Table S1.** 2019 ESC algorithm.

**Additional file 2: Table S2.** Bova score.

**Additional file 3: Figure S1.** Thrombus location.

**Additional file 4: Figure S2.** Cardiac measurement diameter.

## Data Availability

The datasets used and/or analyzed during the current study are available from the corresponding author on reasonable request.

## Abbreviations

ESC: European Society of Cardiology
PE: Pulmonary embolism
CT: Computed tomography
c-Tn I: Cardiac troponin I
NT-pro BNP: N-terminal pro-brain natriuretic peptide
RV: Right ventricle
LV: Left ventricle
RA: Right atrium
LA: Left atrium
CPA: Central pulmonary artery
CART: Classification and regression tree
ROC: Receiver-operating characteristic curve
AUC: Area under the curve
OR: Odds ratio
CI: Confidence interval
PPV: Positive predictive value
NPV: Negative predictive value
DCA: Decision curve analysis
C-index: Concordance index
PH: Pulmonary hypertension

## References

[1] Konstantinides SV, Meyer G, Becattini C, Bueno H, Geersing GJ, Harjola VP, Huisman MV, Humbert M, Jennings CS, Jimenez D (2019). 2019 ESC Guidelines for the diagnosis and management of acute pulmonary embolism developed in collaboration with the European Respiratory Society (ERS): the Task Force for the diagnosis and management of acute pulmonary embolism of the European Society of Cardiology (ESC). Eur Respir J.

[2] Konstantinides SV, Torbicki A, Agnelli G, Danchin N, Fitzmaurice D, Galie N, Gibbs JS, Huisman MV, Humbert M, Kucher N (2014). 2014 ESC guidelines on the diagnosis and management of acute pulmonary embolism. Eur Heart J.

[3] Vedovati MC, Cimini LA, Pierpaoli L, Vanni S, Cotugno M, Pruszczyk P, Di Filippo F, Stefanone V, Torrecillas LG, Kozlowska M (2020). Prognostic value of respiratory index in haemodynamically stable patients with acute pulmonary embolism: the Respiratory Index model study. Eur Heart J Acute Cardiovasc Care.

[4] Lyhne MD, Schultz JG, Kramer A, Mortensen CS, Nielsen-Kudsk JE, Andersen A (2020). Right ventricular adaptation in the critical phase after acute intermediate-risk pulmonary embolism. Eur Heart J Acute Cardiovasc Care.

[5] Kang DK, Thilo C, Schoepf UJ, Barraza JM, Nance JW, Bastarrika G, Abro JA, Ravenel JG, Costello P, Goldhaber SZ (2011). CT signs of right ventricular dysfunction: prognostic role in acute pulmonary embolism. JACC Cardiovasc Imaging.

[6] Lu MT, Demehri S, Cai T, Parast L, Hunsaker AR, Goldhaber SZ, Rybicki FJ (2012). Axial and reformatted four-chamber right ventricle-to-left ventricle diameter ratios on pulmonary CT angiography as predictors of death after acute pulmonary embolism. AJR Am J Roentgenol.

[7] Beenen LFM, Bossuyt PMM, Stoker J, Middeldorp S (2018). Prognostic value of cardiovascular parameters in computed tomography pulmonary angiography in patients with acute pulmonary embolism. Eur Respir J.

[8] Jimenez D, Kopecna D, Tapson V, Briese B, Schreiber D, Lobo JL, Monreal M, Aujesky D, Sanchez O, Meyer G (2014). Derivation and validation of multimarker prognostication for normotensive patients with acute symptomatic pulmonary embolism. Am J Respir Crit Care Med.

[9] Hobohm L, Becattini C, Konstantinides SV, Casazza F, Lankeit M (2020). Validation of a fast prognostic score for risk stratification of normotensive patients with acute pulmonary embolism. Clin Res Cardiol.

[10] Fernandez C, Bova C, Sanchez O, Prandoni P, Lankeit M, Konstantinides S, Vanni S, Fernandez-Golfin C, Yusen RD, Jimenez D (2015). Validation of a model for identification of patients at intermediate to high risk for complications associated with acute symptomatic pulmonary embolism. Chest.

[11] Pinsky MR (2016). The right ventricle: interaction with the pulmonary circulation. Crit Care.

[12] Meinel FG, Nance JW, Schoepf UJ, Hoffmann VS, Thierfelder KM, Costello P, Goldhaber SZ, Bamberg F (2015). Predictive value of computed tomography in acute pulmonary embolism: systematic review and meta-analysis. Am J Med.

[13] Bax S, Jacob J, Ahmed R, Bredy C, Dimopoulos K, Kempny A, Kokosi M, Kier G, Renzoni E, Molyneaux PL (2020). Right ventricular to left ventricular ratio at CT pulmonary angiogram predicts mortality in interstitial lung disease. Chest.

[14] Jaff MR, McMurtry MS, Archer SL, Cushman M, Goldenberg N, Goldhaber SZ, Jenkins JS, Kline JA, Michaels AD, Thistlethwaite P (2011). Management of massive and submassive pulmonary embolism, iliofemoral deep vein thrombosis, and chronic thromboembolic pulmonary hypertension. Circulation.

[15] Jia D, Li XL, Zhang Q, Hou G, Zhou XM, Kang J (2019). A decision tree built with parameters obtained by computed tomographic pulmonary angiography is useful for predicting adverse outcomes in non-high-risk acute pulmonary embolism patients. Respir Res.

[16] Barker AJ, Roldan-Alzate A, Entezari P, Shah SJ, Chesler NC, Wieben O, Markl M, Francois CJ (2015). Four-dimensional flow assessment of pulmonary artery flow and wall shear stress in adult pulmonary arterial hypertension: results from two institutions. Magn Reson Med.

[17] Zuin M, Rigatelli G, Zonzin P, Casazza F, Roncon L (2017). Saddle pulmonary embolism in hemodynamically stable patients: to lyse or not to lyse? An issue in no guidelines land. Eur J Intern Med.

[18] Aribas A, Keskin S, Akilli H, Kayrak M, Erdogan HI, Guler I, Yildirim O, Bekci TT (2014). The use of axial diameters and CT obstruction scores for determining echocardiographic right ventricular dysfunction in patients with acute pulmonary embolism. Jpn J Radiol.

[19] Moons KG, Altman DG, Reitsma JB, Ioannidis JP, Macaskill P, Steyerberg EW, Vickers AJ, Ransohoff DF, Collins GS (2015). Transparent reporting of a multivariable prediction model for Individual Prognosis or Diagnosis (TRIPOD): explanation and elaboration. Ann Intern Med.

[20] Langendijk JA, Slotman BJ, van der Waal I, Doornaert P, Berkof J, Leemans CR (2005). Risk-group definition by recursive partitioning analysis of patients with squamous cell head and neck carcinoma treated with surgery and postoperative radiotherapy. Cancer.

[21] Wolff RF, Moons KGM, Riley RD, Whiting PF, Westwood M, Collins GS, Reitsma JB, Kleijnen J, Mallett S, Groupdagger P (2019). PROBAST: a tool to assess the risk of bias and applicability of prediction model studies. Ann Intern Med.

[22] Lei Z, Li J, Wu D, Xia Y, Wang Q, Si A, Wang K, Wan X, Lau WY, Wu M, Shen F (2016). Nomogram for preoperative estimation of microvascular invasion risk in hepatitis B virus-related hepatocellular carcinoma within the Milan criteria. JAMA Surg.

[23] Aviram G, Soikher E, Bendet A, Shmueli H, Ziv-Baran T, Amitai Y, Friedensohn L, Berliner S, Meilik A, Topilsky Y (2016). Prediction of mortality in pulmonary embolism based on left atrial volume measured on CT pulmonary angiography. Chest.

[24] Wu S, Zheng J, Li Y, Yu H, Shi S, Xie W, Liu H, Su Y, Huang J, Lin T (2017). A radiomics nomogram for the preoperative prediction of lymph node metastasis in bladder cancer. Clin Cancer Res.

[25] DeLong ER, DeLong DM, Clarke-Pearson DL (1988). Comparing the areas under two or more correlated receiver operating characteristic curves: a nonparametric approach. Biometrics.

[26] Grifoni S, Olivotto I, Cecchini P, Pieralli F, Camaiti A, Santoro G, Conti A, Agnelli G, Berni G (2000). Short-term clinical outcome of patients with acute pulmonary embolism, normal blood pressure, and echocardiographic right ventricular dysfunction. Circulation.

[27] Harjola VP, Mebazaa A, Celutkiene J, Bettex D, Bueno H, Chioncel O, Crespo-Leiro MG, Falk V, Filippatos G, Gibbs S (2016). Contemporary management of acute right ventricular failure: a statement from the Heart Failure Association and the Working Group on Pulmonary Circulation and Right Ventricular Function of the European Society of Cardiology. Eur J Heart Fail.

[28] Amaral R, Bousquet J, Pereira AM, Araujo LM, Sa-Sousa A, Jacinto T, Almeida R, Delgado L, Fonseca JA (2019). Disentangling the heterogeneity of allergic respiratory diseases by latent class analysis reveals novel phenotypes. Allergy.

[29] Santos AR, Freitas P, Ferreira J, Oliveira A, Goncalves M, Faria D, Bicho Augusto J, Simoes J, Santos A, Gago M (2020). Risk stratification in normotensive acute pulmonary embolism patients: focus on the intermediate-high risk subgroup. Eur Heart J Acute Cardiovasc Care.

[30] Sanchez O, Trinquart L, Caille V, Couturaud F, Pacouret G, Meneveau N, Verschuren F, Roy PM, Parent F, Righini M (2010). Prognostic factors for pulmonary embolism: the prep study, a prospective multicenter cohort study. Am J Respir Crit Care Med.

[31] Sugiura T, Tanabe N, Matsuura Y, Shigeta A, Kawata N, Jujo T, Yanagawa N, Sakao S, Kasahara Y, Tatsumi K (2013). Role of 320-slice CT imaging in the diagnostic workup of patients with chronic thromboembolic pulmonary hypertension. Chest.

[32] Roeleveld RJ, Marcus JT, Faes TJ, Gan TJ, Boonstra A, Postmus PE, Vonk-Noordegraaf A (2005). Interventricular septal configuration at mr imaging and pulmonary arterial pressure in pulmonary hypertension. Radiology.

[33] Lankeit M (2017). Always think of the right ventricle, even in "low-risk" pulmonary embolism. Eur Respir J.

[34] Cote B, Jimenez D, Planquette B, Roche A, Marey J, Pastre J, Meyer G, Sanchez O (2017). Prognostic value of right ventricular dilatation in patients with low-risk pulmonary embolism. Eur Respir J.

