# Supplementary material for: Developing a scoring tool to estimate the risk of deterioration for normotensive patients with acute pulmonary embolism on admission
Source: Respir Res. 2021 Jan 6;22:9. doi: 10.1186/s12931-020-01602-x (PMC7788965; doi:10.1186/s12931-020-01602-x)
Supplement: Supplementary file 1 — Additional file 1: Table S1. 2019 ESC algorithm. [file 12931_2020_1602_MOESM1_ESM.docx]

**Table S1 2019 ESC algorithm**

| Risk | | simply PE severity index≥1 | RV dysfunction | Elevated laboratory data |
| --- | --- | --- | --- | --- |
| Intermediate-risk | Intermediate-high | Positive | Positive | Positive |
|  | Intermediate-low | Positive | One (or none) positive | |
| Low-risk | | Negative | Negative | Negative |

*ESC* European Society of Cardiology; *PE* pulmonary embolism; *RV* right ventricular
