# Supplementary material for: Developing a scoring tool to estimate the risk of deterioration for normotensive patients with acute pulmonary embolism on admission
Source: Respir Res. 2021 Jan 6;22:9. doi: 10.1186/s12931-020-01602-x (PMC7788965; doi:10.1186/s12931-020-01602-x)
Supplement: Supplementary file 2 — Additional file 2: Table S2. Bova score. [file 12931_2020_1602_MOESM2_ESM.docx]

**Table S2 Bova score**

| Parameter | Scores |
| --- | --- |
| c-Tn I＞0.05ug/L | 2 |
| RV dysfunction | 2 |
| Heart rate≥110beats/min | 1 |
| systolic pressure(90-100mmHg) | 2 |
| Low risk：0-2 scores；  Intermediate-low risk：3-4 scores；  Intermediate-high risk:> 4scores； | |

*c-Tn I* Cardiac troponin I
