# Supplementary material for: Developing a scoring tool to estimate the risk of deterioration for normotensive patients with acute pulmonary embolism on admission
Source: Respir Res. 2021 Jan 6;22:9. doi: 10.1186/s12931-020-01602-x (PMC7788965; doi:10.1186/s12931-020-01602-x)
Supplement: Supplementary file 3 — Additional file 3: Figure S1. Thrombus location. [file 12931_2020_1602_MOESM3_ESM.docx]

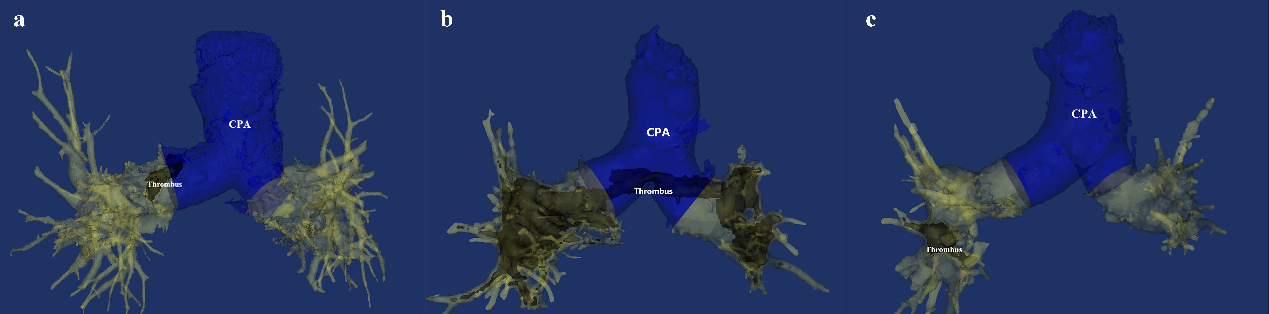


Figure S1. Thrombus location

1. CPA embolism;
2. Saddle-CPA embolism;
3. non-CPA embolism;

*CPA*, central pulmonary artery
