# Supplementary material for: Developing a scoring tool to estimate the risk of deterioration for normotensive patients with acute pulmonary embolism on admission
Source: Respir Res. 2021 Jan 6;22:9. doi: 10.1186/s12931-020-01602-x (PMC7788965; doi:10.1186/s12931-020-01602-x)
Supplement: Supplementary file 4 — Additional file 4: Figure S2. Cardiac measurement diameter. [file 12931_2020_1602_MOESM4_ESM.docx]

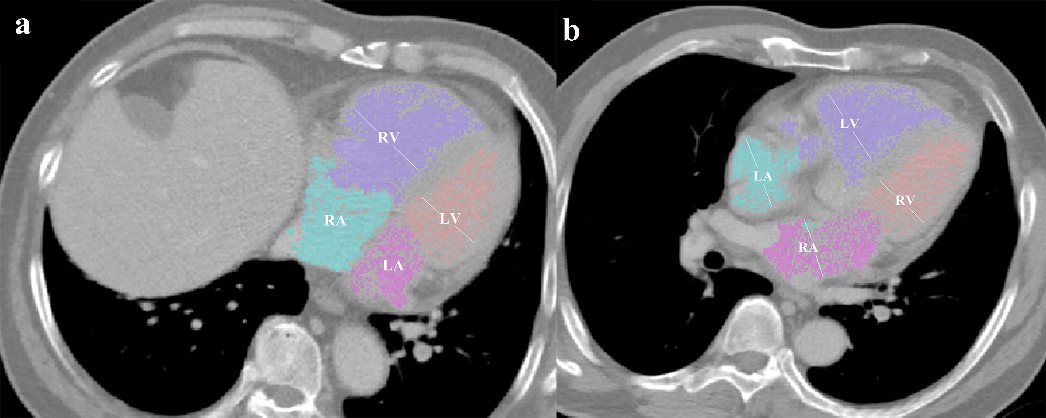


Figure S2. Cardiac measurement diameter

1. Short-axis plane;
2. Four-chamber view;

*RV* right ventricle; *LV* left ventricle; *RA* right atrium; *LA* left atrium
